# Beta cell function, insulin resistance and vitamin D status among type 2 diabetes patients in Western Kenya

**DOI:** 10.1038/s41598-021-83302-0

**Published:** 2021-02-18

**Authors:** Jamil Said, David Lagat, Allan Kimaina, Chrispine Oduor

**Affiliations:** 1 Department of Human Anatomy, Moi University School of Medicine, Eldoret, Kenya; 2 Department of Medicine, Moi University School of Medicine, Eldoret, Kenya; 3 The Academic Model Providing Access To Healthcare (AMPATH), Eldoret, Kenya

**Keywords:** Endocrine system and metabolic diseases, Diabetes, Type 2 diabetes

## Abstract

Serum vitamin D status exerts effects on glucose-insulin-homeostatic states underlying Diabetes-Mellitus, Type 2 (T2DM). This has been described in white and Asian population where low Vitamin D levels predicted future impairments in beta cell function and worsening of insulin resistance. This study aimed to examine the relationship between serum vitamin D, insulin resistance and beta cell function in a sub population of black Kenyan T2DM patients. The primary objective was to determine the levels of serum 25 hydroxy (25-OH) vitamin D, and estimate the insulin resistance, and beta cell function among T2DM patients at Moi Teaching and Referral Hospital (MTRH). This was a cross sectional study. 124 T2DM patients attending the MTRH Diabetes clinic between February and May 2016 were enrolled. Patients on insulin therapy and/or thiazolidinediones were excluded. Anthropometric, clinical and demographic data was obtained. Samples were drawn for estimation of serum 25-OH vitamin D, fasting insulin levels and fasting blood glucose levels. HOMA (Homeostatic model of assessment) model was used to estimate Beta cell secretion (HOMA-B) and insulin resistance (HOMA-IR); while the Disposition index {(DI) hyperbola product of insulin sensitivity (1/HOMA-IR) and beta cell secretion} was used to estimate the beta cell function. The relationships between serum vitamin D, insulin resistance and beta cell function were explored using a linear regression model. The study participants had a mean age of 56.2 (± 9.2) years, and a mean BMI of 26.9 kg/m^2^ (4.3). Forty nine percent (n = 61) were males. Vitamin D deficiency was present in 71.1% (n = 88) of the respondents. Relatively low levels of insulin resistance and higher levels of beta cell dysfunction were observed {median HOMA-IR of 2.3 (0.7, 6.5) and Disposition Index (DI) of 25.5 (14.3, 47.2)}. Vitamin D levels exhibited a low positive correlation with DI [r = 0.22 (95% CI: 0.03, 0.37)], but was not significantly correlated with HOMA-IR [r = 0.07(95% CI: − 0.11, 0.25)]. These results indicate that beta cell dysfunction rather than insulin resistance as the predominant defect among black T2DM patients seeking care at the MTRH diabetes clinic. Vitamin D deficiency is also prevalent among them and exhibits a low positive correlation with beta cell dysfunction. There was no correlation observed between Vitamin D deficiency and insulin resistance.

## Introduction

Almost one billion people worldwide are reported to have either vitamin D deficiency or insufficiency^1^; the prevalence of which varies with ethnic background, geographical location, sunlight exposure, age, and obesity^2,3^.

Co-emerging with the increasing prevalence of hypovitaminosis D, is an association between prevalent type 2 diabetes and vitamin D deficiency^4^; with some studies demonstrating a predictable relationship between Vitamin D levels on one hand, and future glycaemic state and insulin resistance on the other hand^5–7^. A direct relationship between vitamin D and hyperglycaemia, that is influenced by increase in age, has also been reported^8^.

Evidence from both in-vivo and in vitro studies suggest extra-skeletal effects of low vitamin D contributing towards attenuated insulin action and secretion^1^; (^9^; with further demonstration of an inverse relationship between hypovitaminosis D and glycaemic control^10,11^. And contrary to traditional belief, Vitamin D deficiency is being increasingly described among people with dark skinned complexion (^12^.

There are however, also conflicting results from other studies, where no association between T2DM and hypovitaminosis D was found^13,14^; with some of the studies only demonstrating a very weak relationship between the two entities^15,16^. This is despite there being data suggesting involvement of vitamin D in the pathogenesis of type 2 diabetes mellitus^11,17,18^.

In light of the potential existence of an unknown relationship between serum Vitamin D levels and the T2DM state in Kenya, our study sought to bridge the existing knowledge gap by assessing the prevalence of hypovitaminosis D among T2DM patients in Kenya and assessing the association thereof with insulin resistance and beta cell function.

## Methodology

### Study setting

The study was carried out at the diabetes outpatient clinic of Moi Teaching and Referral Hospital (MTRH). MTRH is the second largest public hospital in Kenya and serves as a referral facility for western Kenya, some parts of Eastern Uganda, South Sudan and Tanzania. It has a catchment population of approximately 16.24 million people^19^. Daily, approximately 20 patients with diabetes mellitus type 2 are managed at the hospital. The hospital also serves as a teaching facility for medical undergraduate and post-graduate students.

MTRH is located along Nandi road in Eldoret town, within the larger Uasin Gishu county. The town is located between a latitude of 0.514277 and a longitude of 35.269779 (DMS Lat 0° 30′51.3972″ N and DMS Long 35° 16′11.2044″ E) elevated at an altitude of 2071 m (6795 feet) above sea level.

### Study population

The study population included all patients with type 2 diabetes mellitus (T2DM) who sought outpatient diabetes care services at the Moi Teaching and Referral Hospital.

### Study design

This was a cross sectional study that involved examination of all T2DM patients between the months of February and May 2016. Clinical, laboratory and demographic characteristics of the patients recruited to the study were observed.

### Sample size

In order to be 95% sure with a probability of 80% of the correlation coefficient between serum vitamin D and insulin resistance among type 2 diabetic participants, we estimated the sample size with the assumption that the correlation between the two variables is moderately negative: − 0.25. At the time of sample size calculation there weren’t any published studies on an African population with diabetes; as a result, the assumed correlation was arrived at based on a study done among the Chinese participants with normal glucose^20^ that reported a correlation coefficient of − 0.18. Using the following Fisher’s Z transformation formula^21^.
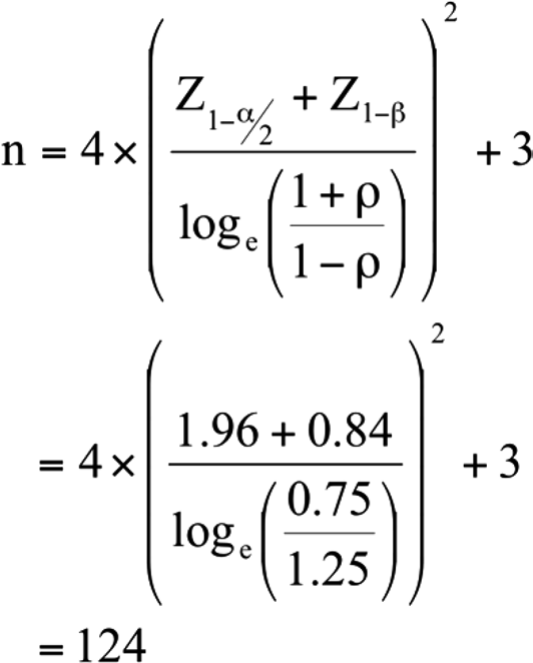
 where $\rho$ is the population correlation coefficient, assumed − 0.25, $\beta$ is the type II error, assumed 20%, while $\alpha$ is the type I error, assumed to be 5%.

Therefore, the required study size was 124.

### Eligibility criteria

#### Inclusion criteria

- Diagnosed cases of diabetes mellitus type 2 on follow up at the MTRH diabetes clinic.
- Individuals of 18 years of age and above.

#### Exclusion criteria

- Evidence of diminished liver synthetic function based on low serum Albumin levels of < 35 g/L.
- Evidence of renal disease with diminished Glomerular Filtration rate (GFR) of less than 60mls/min/1.73m2.
- Patients on exogenous Insulin or Thiazolidinedione treatment.
- Patients on vitamin D supplements.

### Materials and procedure

#### Subjects

Potential study participants were screened from the MTRH diabetes outpatient clinic and referred to the study recruitment site which was located at an adjacent room next to the main diabetes clinic for recruitment if they met the eligibility criteria. A sampling frame of eligible study participants was created during each clinic day, upon which a computer-generated random sample was obtained based on sampling ratio of 15:20 (15 random selections for every 20 eligible subjects). (The sampling and subject recruitment details are further described in Supplementary Appendix 1).

#### Measurements

Eight to fourteen hours of fasting blood samples were collected for the estimation of serum insulin levels, whole blood glucose levels and serum 25(OH) Vitamin D levels. Details on the precise estimation method of beta cell function and insulin sensitivity; as well as the other biochemical parameters tested are further explained in Supplementary Appendix 2.

### Data collection and management

#### Data collection

An interviewer-administered structured questionnaire was used to collect data on demographic characteristics, and detailed medical history of the participants. Laboratory data and anthropometric measurements were obtained and entered in the data collection forms. Medical records were also reviewed, and other relevant clinical and laboratory data were obtained and entered in the data collection forms.

#### Data analysis and presentation

Data analysis was done using software for statistical computing known as R^22^. Relationships between vitamin D, beta cell function, and insulin resistance, were explored using scatter plots and loess curves. The relationships were found to be nonlinear, so natural logarithm of beta cell function and insulin resistance were calculated.

The association between continuous variables (e.g. insulin sensitivity, beta cell function, and disposition index) and categorical variables (e.g. education levels, and vitamin D levels) were assessed using Kruskal–Wallis test, while the association between categorical variables was assessed using Pearson’s Chi Square test. Fisher’s exact test was used whenever the Chi Square assumptions were violated. Linear regression model was used to assess the relationship between vitamin D on beta cell function and insulin resistance adjusting for confounding variables. Pearson’s rank correlation coefficients were used to assess the relationship between vitamin D, insulin resistance and disposition index.

### Ethical considerations

Approval was sought from the Moi university college of health sciences and Moi teaching and referral hospital Institutional Research Ethics Committee (IREC) before study commencement. Permission to conduct the study was also further obtained from the management of Moi Teaching and Referral Hospital. All study methods were performed in accordance with good clinical practice guidelines and regulations.

All study subjects provided informed consent to participate in the study. Further details pertaining to ethics, data safety management and confidentiality are elaborated in Supplementary Appendix 3.

## Results

**Figure 1:**
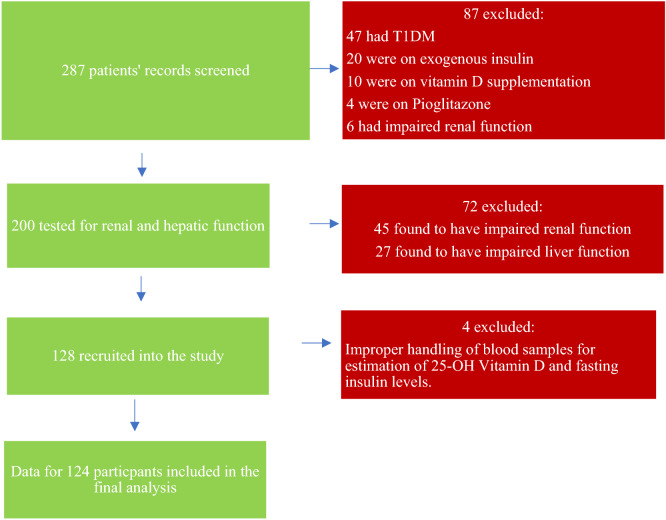
Patient recruitment schema flow chart.

A total of 287 patients were screened for entry into the study. 159 were excluded, as indicated in the recruitment flow chart in Fig. 1, leaving a total of 128 participants who were then included in the final analysis. Their demographic, clinical, treatment and outcome characteristics were as shown in Tables 1, 2, 3 and 4.

**Table 1:** Demographic characteristics of the study participants.

Variable	Mean (SD) or n (%)
Age	56.2 (9.2)
Male	63 (49.2%)
Level of education*	
None	15 (12.1%)
Primary	71 (57.3%)
Secondary	33 (26.6%)
Tertiary (College/University)	5 (4.0%)

*n = 124.

**Table 2:** Clinical characteristics of the study participants.

Variable	Mean (SD) or Median (IQR)
Creatinine (mmol/L)	62.5 (53.0, 71.0)
EGFR (CKD–EPI) ml/min/1.73m^2^	115.8 (105.1, 124.1
Albumin	43.3 (3.5)
Weight (kg)	74.0 (11.4)
Height (cm)	166.2 (6.9)
BMI (kg/m^2^)	26.9 (4.3)
< 18.5	2 (1.6%)
18.5–25.0	38 (29.7%)
25.0–30.0	65 (50.8%)
> 30.0	23 (18.0%)
Systolic blood pressure (SBP) ^+^ mm Hg	120.0 (114.8, 130.3)
Diastolic blood pressure (DBP) ^+^ mm Hg	78.0 (68.8, 89.0)
SBP > 140 mm Hg|DBP > 90 mm Hg	38 (30.6%)
Insulin IU	8.8 (2.7, 22.9)
Fasting Blood Sugar	6.3 (4.8, 7.3)
Vitamin D	16.0 (1.6, 21.1)

^+^n = 124.

**Table 3:** Treatment characteristics.

Treatment	n (%)
Diet	21 (16.4%)
Biguanides–Metformin	98 (76.6%)
Sulphonylureas	49 (38.3%)
Types of sulphonylureas (n = 49)	
Glibenclamide	47 (95.9%)
Gliclazide	1 (2.0%)
Glimepride	1 (2.0%)
Thiazide Diuretics–HCTz	34 (26.6%)
ACE inhibitors–Enarapril	19 (14.8%)
Angiotensin II receptor blocker–Losartan	11 (8.6%)
Platelet aggregation inhibitor–JASA	3 (2.3%)
Calcium channel blockers	17 (13.3%)
Types of calcium channel blockers	
Amlodipine	5 (29.4%)
Nifedipine	12 (70.6%)
Statin–Atorvastatin	9 (7.0%)
Beta blocker–Carvedilol	1 (0.8%)
Anti-depressants anxiolytics–Amitriptyline	3 (2.3%)
Anti-retroviral Therapy (ARV)	1 (0.8%)
Coumarin–warfarin	1 (0.8%)

**Table 4:** Outcome characteristics.

Variables	Median (IQR)
Beta cell function (n = 120)	84.9 (54.8, 160.3)
Insulin resistance (n = 120)	2.3 (0.7, 6.5)
Disposition Index (n = 120)	25.5 (14.3, 47.2)

The mean (SD) age was 56.2 (0.2) years with a minimum and a maximum of 36.0 and 86.0 respectively. Half, 63 (49.2%), of the participants were male. Over half 71 (57.3%), and 33 (26.6%) attained primary and secondary levels of education respectively. More than 10% had no formal education.

The median creatinine was 62.5 (IQR: 53.0, 71.0) micromole per litre. The median estimated glomerular filtration (Chronic Disease Epidemiology Collaboration) was 115.8 (IQR: 105.1, 124.1) mL/min per 1.73 m^2^. The average albumin was 43.3 (SD: 3.5) g/dL.

The average weight was 74.0 (SD: 11.4) kilograms with an average BMI of 26.9 (SD: 4.3) kg/m^2^.

The median SBP and DBP were 120.0 (IQR: 114.8, 130.3) mm Hg and 78.0 (IQR: 68.8, 89.0) mm Hg respectively. One third of the sample were hypertensive (SBP > 140 mm Hg|DBP > 90 mm Hg) 38 (30.6%).

The median insulin level was 0.4 (IQR: 0.1, 0.9) ng/dL, and the fasting blood sugar was 6.3 (IQR: 4.8, 7.3) mmol/L.

Median vitamin D levels was 16.0 (IQR: 1.6, 21.1).

One sixth of the participants (16.4%) were on diet. Three quarters (76.6%) of the participants were on metformin. Sulphonylureas such as glibenclamide, gliclazide, and glimepride were in use by 49 (38.2%) participants. HCTz was used by 33 (25.8%) of the participants, and enalapril was being used by 19 (14.8%). Only one participant was on carvedilol. There was one participant on anti-retroviral therapy, and one was on warfarin.

**Figure 2:**
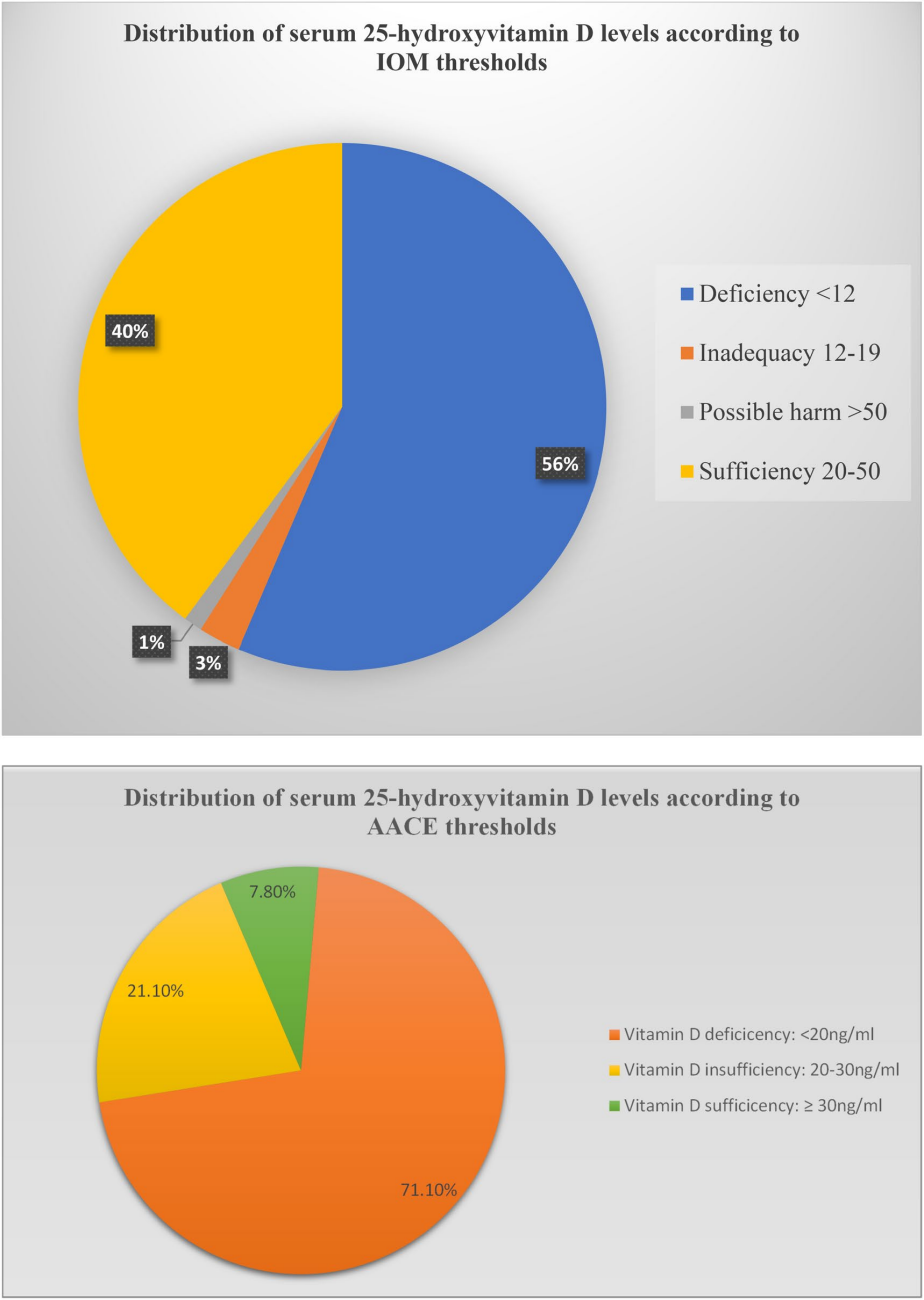
Comparison of distribution of serum 25-hydroxyvitamin D levels according to IOM (Institute Of Medicine) and AACE (American Academy of Clinical Endocrinology) thresholds.

There was a total of 118 (92.2%, 95% CI: 87.5, 96.9) participants with hypovitaminosis D. The proportions of Vitamin D sufficiency, insufficiency and deficiency are further highlighted in Fig. 2.

The median beta cell function, insulin resistance, and disposition index were 84.9 (IQR: 54.8, 160.3) ng/ml, 2.3 (IQR: 0.7, 6.5), and 25.5 (14.3, 47.2) respectively.

**Figure 3:**
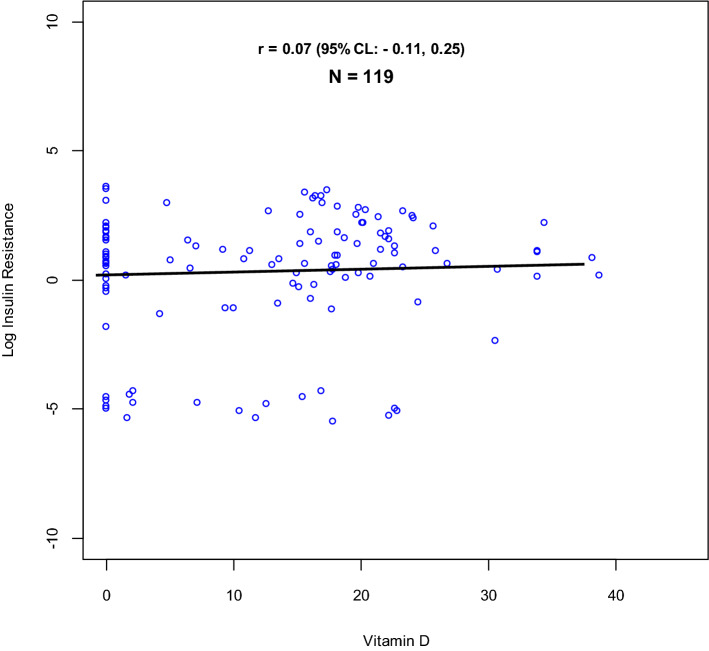
Relationship between vitamin D and log insulin resistance.

**Figure 4:**
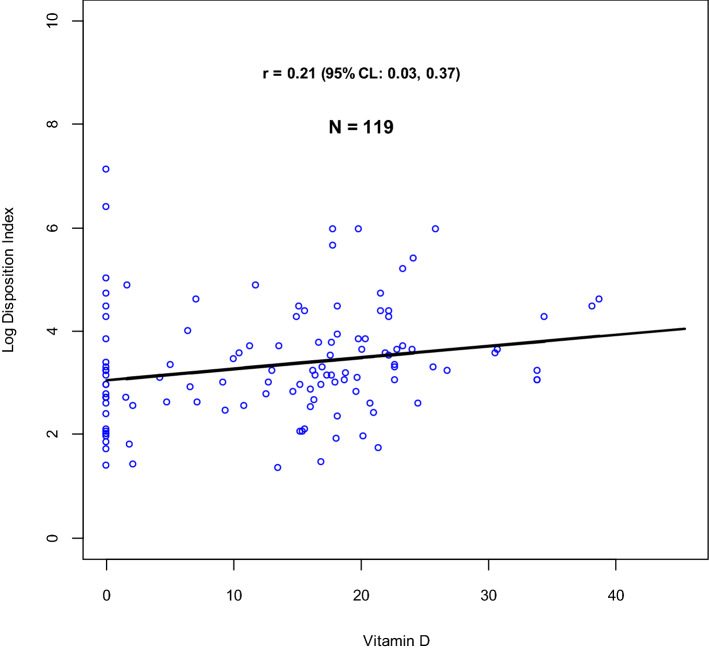
Relationship between vitamin D and log disposition index.

The relationship between insulin resistance, and disposition index with vitamin D were studied using scatter plots and linear regression models as shown in Figs. 3 and 4.

There was no linear relationship between (natural logarithm of) insulin resistance and serum 25 hydroxyvitamin D levels even after adjusting for BMI r = 0.07(95% CI: − 0.11, 0.25).

**Figure 5:**
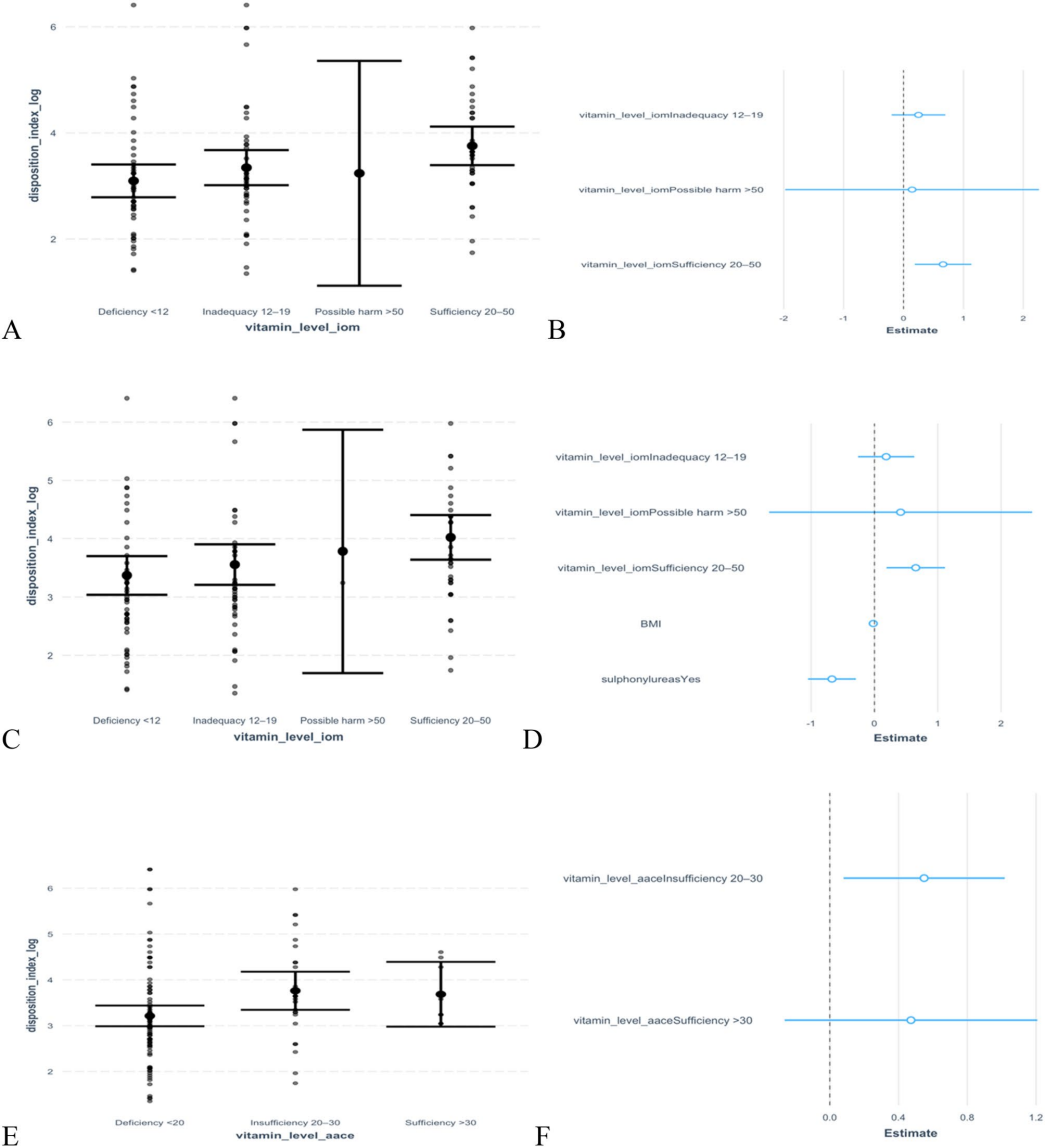

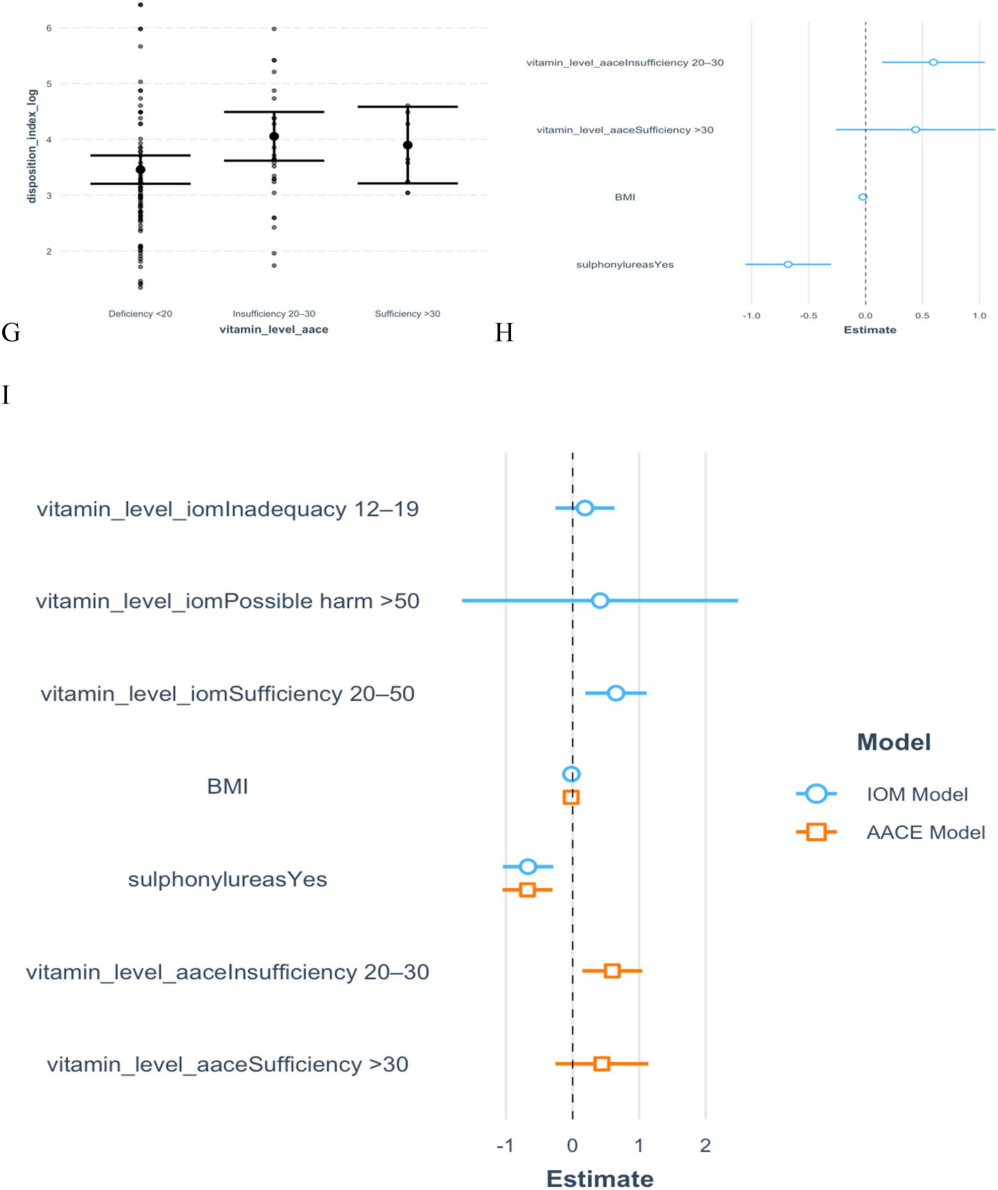
Adjusted and un-adjusted relationship between vitamin D and log of disposition index, with comparison between IOM and AACE Vitamin D thresholds. (**A**) Box plots showing un-adjusted linear regression correlation between Vitamin D levels (categorised according to IOM thresholds) and log of disposition index. (**B**) Forest plot showing un-adjusted linear regression correlation between Vitamin D levels (categorised according to IOM thresholds) and log of disposition index. (**C**) Box plots showing linear regression correlation between Vitamin D levels (categorised according to IOM thresholds) and log of disposition index, adjusted for BMI and sulfonylurea use. (**D**) Forest plot showing linear regression correlation between Vitamin D levels (categorised according to IOM thresholds) and log of disposition index, adjusted for BMI and sulfonylurea use. (**E**) Box plots showing un-adjusted linear regression correlation between Vitamin D levels (categorised according to AACE thresholds) and log of disposition index. (**F**) Forest plot showing un-adjusted linear regression correlation between Vitamin D levels (categorised according to AACE thresholds) and log of disposition index. (**G**) Box plots showing linear regression correlation between Vitamin D levels (categorised according to AACE thresholds) and log of disposition index, adjusted for BMI and sulfonylurea use. (**H**) Forest plot showing linear regression correlation between Vitamin D levels (categorised according to AACE thresholds) and log of disposition index, adjusted for BMI and sulfonylurea use. (**I**) Forest plot comparing linear regression correlation between Vitamin D levels (categorised according to both IOM and AACE thresholds) and log of disposition index, after adjustment for BMI and sulfonylurea use.

Evidently from the data, there is a weak positive correlation between (natural logarithm of) disposition index and vitamin D, r = 0.21 (95% CI: 0.03, 0.37). An exploration of the association between vitamin D and beta cell function, based on AACE and IOM vitamin D thresholds, is further highlighted in Fig. 5.

The predictors of disposition index were vitamin D and use of sulphonylureas as is further highlighted in Table 5. After adjusting the effect of vitamin D for use of sulphonylureas, the magnitude of the estimate increased, 0.22 (95% CI: 0.03, 0.40). Participants who were using sulphonylureas were significantly associated with low levels of disposition index, − 0.48 (95% CI: − 0.88, − 0.09).

**Table 5:** Effect of vitamin D on disposition index adjusting for confounding variables.

Variable	Unadjusted estimate (95% CI)	Adjusted estimate (95% CI)
Vitamin D	0.02 (0.00, 0.04)	**0.22 (0.03, 0.40)**
Sulphonylureas	− 0.49 (− 0.89, − 0.09)	** − 0.48 (− 0.88, − 0.09)**
Sample Size		119

## Discussion

This study found a high prevalence of hypovitaminosis D among type 2 Diabetes participants. The prevalence of hypovitaminosis D was 92.2% (95% CI 87.5–96.9), where vitamin D deficiency was 71.1% when analysed based on AACE guideline thresholds^23^. Whereas using analysis based on IOM guideline thresholds, prevalence of hypovitaminosis D was 71.3% (95% CI 62.9–78.4) and vitamin D deficiency 39.5%^23^.

All participants were of indigenous local African ethnicity, and all of them lived within a 100 km radius of the Moi Teaching and Referral Hospital (MTRH), an area located 57.31 Kilo Meters (KM) North of the equator, and like the rest of Kenya and Eastern Africa, receives sufficient sun light all year round.

To the best of our knowledge, this is the first study of its kind to be conducted in Kenya and probably the second within Sub Saharan Africa, after Fondjo et al.'s^24^ case control study that was conducted in Ghana. In comparison, Fondjo et al.’s^24^ study reported relatively higher proportions of vitamin D deficiency among their type 2 diabetes cases (92.4% vs 71.1%), based on a cut off threshold of 20 ng/ml.

We settled for a sufficiency threshold of 30 ng/ml which is in keeping with the recommendations of the endocrine society^25^, the international osteoporosis foundation^26^ and the American Geriatric Society (2014); and assumed a baseline healthy population reference that falls above this sufficiency limit. Our assumption was largely informed by findings from Luxwolda et al.’s study^27^, where the mean vitamin D status of healthy non-pregnant indigenous East African adults, drawn from both Nilotic and Bantu subtribes living within 2–4 degrees around the equator, was found to be 42.46 ng/ml.

This study also showed a weak positive correlation between 25 OH Vitamin D concentrations and beta cell function; but no association was observed with insulin resistance.

The inverse association between Vitamin D deficiency and Type 2 Diabetes has been alluded to by findings from various observational studies^28,29^, which have also been recently replicated in some prospective cohort studies; strongest of which are the English Ely cohort study^30^ which followed subjects for a mean period of 10 years; and the Finnish cohort study, which followed subjects for a mean period of 17 years^31^.

Both studies, despite high subject attrition rates which consequently minimized the magnitude of observed effects of associations, demonstrated that low Vitamin D status predicts both future hyperglycaemia and hyperinsulinemia^30,31^.

However; the evidence base for a role of low Vitamin D towards development of type 2 diabetes in subjects of African descent remains weak.

Our findings contrast with those of Fondjo et al.^24^; where similarly using HOMA estimates of beta cell secretion (HOMA-B) and insulin resistance (HOMA-IR), did not find any association, through linear regression analysis, between Vitamin D levels and either beta cell function or insulin resistance among the population that they studied. However, severe and moderate vitamin D deficiency states were found to confer a 22.3 and 12.9-fold increased odds of Type 2 Diabetes respectively^32^. We managed to achieve collinearity between Vitamin D levels and HOMA scores after natural logarithmic transformation of the raw data; Fondjo et al.^24^ on the other hand, seems to have computed linear regressions using the raw data only.

We used basal disposition index (DI), derived from the mathematical product of HOMA estimates of insulin sensitivity (1/HOMA-IR) and basal beta cell secretion (HOMA-B%), as a surrogate measure of beta cell function. The DI assumes a hyperbola relationship between insulin sensitivity and beta cell insulin secretion as has been described by Turner et al.^33^; and thus, provides an estimate of the beta cell function relative to the level of insulin sensitivity; more specifically hepatic insulin sensitivity for that matter.

Since fasting plasma glucose was used in the calculation estimates, which is mainly of hepatic origin^34^, it is likely that the resulting insulin resistance index obtained (calculated by the HOMA IR linear equation) refers to more of hepatic as opposed to peripheral or total body insulin resistance^35^. This measure of hepatic insulin resistance, as has been previously described in the literature^36,37^, can be used to calculate the hepatic disposition index.

Our demonstration of a positive association between 25 OH Vitamin D levels and beta cell function as measured by the DI, does seem to correlate with findings reported by Kodama et al.^38^. In this met-analysis of 74 cohorts comprising 3813 individuals, normal glucose tolerant Africans and East Asians were found to sit along the extreme regions of the hyperbola curve of insulin secretion and insulin resistance; with Africans having predominantly higher baseline insulin secretion and insulin resistance levels. Based on these findings, the authors suggested a profound reduction in insulin secretion as the critical determinant of progression from normal glucose tolerance to Type 2 Diabetes among individuals of African descent.

We found a median HOMA IR of 2.3 (0.7, 6.5). This overlaps with ranges reported among African non-diabetic individuals^39^. However; much lower HOMA IR levels have also been reported^40^. On the other hand significantly higher levels have been found among African individuals with Type 2 Diabetes^41^.

In summary, a high proportion of the Type 2 Diabetes mellitus patients on follow up at the Moi Teaching and Referral hospital have hypovitaminosis D which appears to be inversely associated with beta cell function but not insulin resistance. It also appears that among our Type 2 Diabetes patients, beta cell dysfunction is the predominant feature contributing to dysglyceamia as opposed to insulin resistance. These findings, however, need to be confirmed through prospective studies that use formal methods of assessing insulin resistance, insulin secretion and vitamin D status, such as the hyper-insulinaemic euglycemic clamp, frequently sampled intravenous glucose tolerance test and high-performance liquid chromatography respectively.

## Conclusion

Impaired beta cell dysfunction rather than insulin resistance is the more predominant dysglyceamic defect among T2DM patients seeking care at the MTRH diabetes clinic.

A high proportion of these patients have hypovitaminosis D which appears to be inversely associated with beta cell function but not insulin resistance.

Due to absence of robust evidence of association between glycemia and vitamin D states; we recommend screening for and supplementing Vitamin D deficiency states among our Type 2 Diabetes patients in accordance with international guideline recommendations which are aimed towards skeletal benefit.

## Limitations

This being a cross-sectional study, any causal association between vitamin status and beta cell function or insulin resistance cannot be determined and will need to be explored through a prospective study.

We were also unable to draw accurate comparison between population levels of HOMA-IR insulin resistance and those of Type 2 diabetes cases due to lack of a comparator arm, as well as lack of well-defined population references within the Kenyan and sub-Saharan African context.

## Supplementary Information

Supplementary Information 1.

Supplementary Information 2.

Supplementary Information 3.
