# Supplementary material for: Beta cell function, insulin resistance and vitamin D status among type 2 diabetes patients in Western Kenya
Source: Sci Rep. 2021 Feb 18;11:4084. doi: 10.1038/s41598-021-83302-0 (PMC7892854; doi:10.1038/s41598-021-83302-0)
Supplement: Supplementary file 1 — Supplementary Information 1. [file 41598_2021_83302_MOESM1_ESM.docx]

**Beta Cell Function, Insulin Resistance and Vitamin D Status Among Type 2 Diabetes Patients in Western Kenya**

Dr. Said Jamil AbdulKadir^*1^

*Department of human anatomy, Moi University School of Medicine*

Dr. Lagat David^2^

*Department of Medicine, Moi University School of Medicine*

Kimaina Allan^3^

*The Academic Model Providing Access To Healthcare (AMPATH), Kenya.*

Dr. Oduor Chrispine^4^

*Department of Medicine, Moi University School of Medicine*

*Corresponding author. Correspondence email: jamilalariik@gmail.com

**Supplementary appendix 1**

1. **Sampling Technique**

MTRH was purposively selected because it is a referral center with functional diabetic care medical clinics. Simple random sampling was used to sample potential participants eligible for the study; and this was derived from those who sought care at the hospital’s Diabetes care clinics. On a daily basis, a computer generated simple random sample, based on a 15:20 random selection (15 random samples per cohort of 20 patients), was used to identify subjects from the selected sampling frame. Selected subjects were then provided with a unique identifier code and subsequently enrolled into the study. This continued until the desired sample size was reached.

**1.1 Participant recruitment**

Sampled participants were then approached by the principal investigator or the research assistant for consent to enroll into the study. Only consenting adults were recruited; and thereafter assigned a unique patient identifier code. The identity of each participant was kept confidential, only known to the principal investigator.

Once relevant clinical data, anthropometric measurements and blood samples were obtained, (see further details in the data collection form attached at the appendix section) each of the study participants was requested to return to the MTRH diabetes clinic on the morning of a specified date while on a 10-12 hrs. fasted state. Each subject received reimbursement of their two-way transportation cost to and from the MTRH diabetes clinic, in addition to a morning meal voucher; totaling to a cash value of 200 KSHS. For subjects whose renal and hepatic function indices were missing from their medical charts, further assessment of study inclusion eligibility was done upon renal and hepatic function testing using estimated GFR and serum albumin levels (further estimation details provided in the section below)

1. **Data collection and management**

### **2.1 Data collection**

An interviewer-administered structured questionnaire was used to collect data on demographic characteristics, and detailed medical history of the participants. Laboratory data and anthropometric measurements were obtained and entered in the data collection forms. Medical records were also reviewed, and other relevant clinical and laboratory data were obtained and entered in the data collection forms.

To determine factors that predicts the association between 25 (OH) D, insulin resistance and beta cell function; clinical and metabolic parameters on age, weight, height, medication history, fasting blood glucose levels, fasting insulin levels, serum creatinine levels, serum 25 (OH) D levels and serum albumin levels were obtained.

### **2.2 Data analysis and presentation**

Data analysis was done using software for statistical computing known as R (R core Team, 2016). Categorical variables were summarized as frequencies and the corresponding percentages. Continuous variables were summarized using mean and the corresponding standard deviation if Gaussian assumptions were satisfied otherwise they were summarized using median and the corresponding interquartile range (IQR). Gaussian assumptions were assessed using Shapiro – Wilk test and using graphical methods such as histograms.

Relationships between vitamin D, and beta cell function, insulin resistance, and disposition index were explored using scatter plots and loess curves. The relationships were found to be nonlinear, so natural logarithm of beta cell function, insulin resistance, and disposition index were calculated. This improved the relationship except for an outlier, a participant who had vitamin D level of 55.06. This value was far placed relative to the rest (a closer one being 38.72), so it was excluded from the analysis.

The association between continuous variables (e.g. insulin sensitivity, beta cell function, and disposition index) and categorical variables (e.g. education levels, and vitamin D levels) were assessed using Kruskal-Wallis test, and the association between categorical variables was assessed using Pearson’s Chi Square test. Fisher’s exact test was used whenever the Chi Square assumptions were violated. Linear regression model was used to assess the effect of vitamin D on beta cell function, insulin resistance and disposition index adjusting for confounding variables. The regression coefficients and the corresponding 95% confidence intervals (95% CI) were reported. Pearson’s rank correlation coefficients were used to assess the relationship between vitamin D, insulin resistance and disposition index. The confidence limits for this estimate was computed and presented. Other results were presented using tables.
