# Supplementary material for: Beta cell function, insulin resistance and vitamin D status among type 2 diabetes patients in Western Kenya
Source: Sci Rep. 2021 Feb 18;11:4084. doi: 10.1038/s41598-021-83302-0 (PMC7892854; doi:10.1038/s41598-021-83302-0)
Supplement: Supplementary file 2 — Supplementary Information 2. [file 41598_2021_83302_MOESM2_ESM.docx]

**Beta Cell Function, Insulin Resistance and Vitamin D Status Among Type 2 Diabetes Patients in Western Kenya**

Dr. Said Jamil AbdulKadir^*1^

*Department of human anatomy, Moi University School of Medicine*

Dr. Lagat David^2^

*Department of Medicine, Moi University School of Medicine*

Kimaina Allan^3^

*The Academic Model Providing Access To Healthcare (AMPATH), Kenya.*

Dr. Oduor Chrispine^4^

*Department of Medicine, Moi University School of Medicine*

*Corresponding author. Correspondence email: jamilalariik@gmail.com

**Supplementary appendix 2**

**Measurements:**

Blood samples were collected from a peripheral antecubital vein, and approximately 2mls of whole blood was placed in either of two plain vacutainers which were appropriately labeled either A or B in addition to the patients’ unique identifier code. Vacutainers labeled A were destined for the biochemistry lab for measurements of serum creatinine and serum albumin levels; whereas vacutainers labelled B were destined for the immunology lab. Fasting blood glucose levels were measured on whole blood immediately at the recruitment clinic during a fasted state visit.

*Serum 25(OH) Vitamin D Measurements:*

Total serum 25(OH) vitamin D levels were measured using a Monobind Inc. AccuBind ELISA micro well test system (product code: 9425-300) which provided quantitative measurements of serum 25 (OH) vitamin D through a colorimetric microplate enzyme immunoassay method (further assay details are available in the test insert attached in the appendix section). Vitamin D status was classified according to the following ranges:

Vitamin D status 25 OH Vitamin D

Deficiency <25 nmol/L

Insufficiency 25-75 nmol/L

Sufficiency 75-250 nmol/L

Toxicity >250 nmol/L

Samples destined for serum 25 (OH) vitamin D estimation were collected on plain vacutainers and transported to the MTRH immunology lab within 2 hours of collection. Once at the lab, the samples were examined and registered by one of two research dedicated lab technicians, who were also primarily MTRH staff stationed at the lab. After registration, samples were immediately centrifuged, and serum was separated from the buffy coat; the latter was disposed, whereas the former stored at -40 degrees Celsius for up to 14 days. Serum 25 (OH) vitamin D measurements were run once every two weeks using long ELISA method (further assay details are available in the test insert attached in the appendix section), and the results were entered into a laboratory data sheet and later transferred by the research assistant to each of the subjects’ data collection form.

*Insulin resistance and beta cell function estimation:*

Fasting serum insulin levels were measured using a Monobind Inc. AccuBind ELISA micro well rapid insulin test system (product code: 5825-200) (further assay details are available in the test insert attached in the appendix section) while fasting whole blood capillary glucose levels were measured using an ABOTT free style point of care hexokinase glucometer. Both fasting serum insulin and fasting whole blood capillary glucose measurements were used for the estimation of insulin resistance and Beta cell function using HOMA estimates (HOMA-IR and HOMA-BS) and the disposition index (DI) respectively. The respective calculations are provided by the equations below:

HOMA-IR = (Fasting serum insulin * fasting plasma glucose) / 22.5

HOMA-BS = (20 * fasting serum insulin)/ (fasting plasma glucose – 3.5)

DI – 1/HOMA-IR * HOMA-BS

Samples destined for serum insulin estimation were collected on plain vacutainers and transported to the MTRH immunology lab within 2 hours of collection. Once at the lab, the samples were examined and registered by one of two research dedicated lab technicians, who were also primarily MTRH staff stationed at the lab. After registration, samples were immediately centrifuged, and serum was separated from the buffy coat; the latter was disposed whereas the former as stored at -40 degrees Celsius for up to 14 days. Serum insulin measurements were run once every two weeks using a long ELISA method, and the results were entered into a laboratory data sheet and later transferred by the research assistant to each of the subjects’ data collection form.

Fasting blood glucose levels were entered into each of the participants’ data collection forms immediately upon testing. Testing was done at the recruitment clinic.

*Serum creatinine and serum albumin measurements:*

Serum creatinine and serum albumin levels were measured using a biochemistry analyzer Cobas Integra 400 Plus machine, with creatinine estimation done using the jaffe method. GFR estimates were calculated using the CKD-EPI formula and were used as indicators of renal function. Patients with estimated GFR <60 mls/min/1.73m^2^ and serum albumin levels <35g/l were excluded from the study.
