# Supplementary material for: Beta cell function, insulin resistance and vitamin D status among type 2 diabetes patients in Western Kenya
Source: Sci Rep. 2021 Feb 18;11:4084. doi: 10.1038/s41598-021-83302-0 (PMC7892854; doi:10.1038/s41598-021-83302-0)
Supplement: Supplementary file 3 — Supplementary Information 3. [file 41598_2021_83302_MOESM3_ESM.docx]

**Beta Cell Function, Insulin Resistance and Vitamin D Status Among Type 2 Diabetes Patients in Western Kenya**

Dr. Said Jamil AbdulKadir^*1^

*Department of human anatomy, Moi University School of Medicine*

Dr. Lagat David^2^

*Department of Medicine, Moi University School of Medicine*

Kimaina Allan^3^

*The Academic Model Providing Access To Healthcare (AMPATH), Kenya.*

Dr. Oduor Chrispine^4^

*Department of Medicine, Moi University School of Medicine*

*Corresponding author. Correspondence email: jamilalariik@gmail.com

**Supplementary appendix 3**

**Ethical considerations**

Approval was sought from the Moi university college of health sciences and Moi teaching and referral hospital Institutional Research Ethics Committee (IREC) before study commencement. Permission to conduct the study was also further obtained from the management of Moi Teaching and Referral Hospital. All study methods were performed in accordance with good clinical practice guidelines and regulations.

All the participants were informed about the purpose of the study and politely requested without any coercion, or force or pressure to give a signed written informed consent before participating. Interviewed questions were designed in a culturally sensitive and appropriate manner such that the research objectives were properly addressed while at the same time the respect, dignity, privacy and confidentiality of the participant was always maintained. Data was managed in a confidential manner - stored in locked cabinets and password coded databases. Full data access was restricted to only the principal investigator; while patient consenting was done in a private consultation room. There was no direct financial benefit or compensation for participation in the study. Participants only received reimbursements for breakfast meal and travel costs incurred while attending the MTRH diabetes outpatient clinic on pre-specified dates for collection of fasted samples. Sound clinical judgment was applied in all stages and aspects of this research. No participant was denied usual care if they declined to participate in the study, and those who agreed to participate, could withdraw at any time whenever they so desired.
